# Number of Endoscopic Retrograde Cholangiopancreatography Procedures Required for Short Biliary Cannulation Time

**DOI:** 10.1155/2017/1515260

**Published:** 2017-04-12

**Authors:** Koichiro Mandai, Koji Uno, Yasutoshi Fujii, Takuji Kawamura, Kenjiro Yasuda

**Affiliations:** Department of Gastroenterology, Kyoto Second Red Cross Hospital, 355-5 Haruobi-cho, Kamigyo-ku, Kyoto 602-8026, Japan

## Abstract

*Background.* Several previous studies assessed the competence in endoscopic retrograde cholangiopancreatography (ERCP) using the bile duct cannulation success rate. However, the cannulation time is also important, because a long cannulation time was reported to be a risk factor for post-ERCP pancreatitis. *Aim.* To determine the number of ERCP procedures required for short cannulation time of the bile duct. *Methods.* We retrospectively analyzed 605 ERCP procedures performed for bile duct cannulation in patients with native papilla at our institution between March 2012 and December 2015. The successful procedures were divided into 2 groups: group L and group S (cannulation time > 15 minutes and ≤15 minutes, resp.). An analysis of the relationship among the biliary cannulation time, ERCP experience, and other factors was then conducted. *Results.* Multivariate analysis showed that the ERCP experience of ≤300 procedures (odds ratio, 2.080; 95% confidence interval, 1.337–3.142; *P* = 0.001) and malignant biliary stricture due to pancreatic cancer (odds ratio, 1.912; 95% confidence interval, 1.072–3.412; *P* = 0.028) were found to be significantly associated with a cannulation time of >15 minutes. *Conclusions.* Our findings suggested that an ERCP experience of ≤300 procedures and malignant biliary stricture due to pancreatic cancer were associated with prolonged biliary cannulation time.

## 1. Introduction

The skills for endoscopic retrograde cholangiopancreatography (ERCP) are difficult to obtain. Although an accurate marker of the skills for ERCP is not determined, selective bile duct cannulation is an essential technique for therapeutic ERCP and can be regarded as a surrogate marker of the skills for ERCP.

Several previous studies investigated the number of ERCP procedures required for obtaining the skills for selective bile duct cannulation in patients with native papilla [1, 2]. These studies examined cannulation success rate. We agree with this evaluation method, but we believe that in addition to the success rate, cannulation time is also important, because cannulation time of over 10–15 minutes was reported to be a risk factor for post-ERCP pancreatitis (PEP) [3, 4]. No reports regarding the relationship between bile duct cannulation time and ERCP experience are available.

The present study aimed to examine the number of ERCP procedures required for short cannulation time of the bile duct in patients with native papilla, using the analysis of the relationship among the selective bile duct cannulation time, ERCP experience, and other factors.

## 2. Materials and Methods

We performed a retrospective, case-control study of patients with native papilla treated with ERCP procedures for bile duct cannulation between March 2012 and December 2015 at our institution. A papilla was considered native if sphincterotomy and/or stent placement was not performed previously. A total of 2410 ERCP procedures were performed during the period. We excluded from the analysis the procedures performed in patients with surgically altered gastrointestinal or pancreatobiliary anatomy, ampullary cancer, including intraampullary type, or tumor invasion in the papilla, or if the papilla could not be reached. Considering these criteria, we finally evaluated 605 procedures performed by 23 endoscopists with different levels of ERCP experience. The study was reviewed and approved by the Kyoto Second Red Cross Hospital Institutional Review Board. All study participants provided informed consent.

All procedures were performed with the patients placed in the prone position and under procedural sedation with midazolam and pethidine. Additionally, propofol was administered to patients who showed a poor response to sedation.

Bile duct cannulation was attempted using a conventional ERCP cannula with contrast medium injection in all patients. Cannulation time was defined as the time between the start of advancement of the cannula from the endoscope channel placed in front of the papilla and the confirmation of successful deep bile duct cannulation with a contrast medium injection. If selective bile duct cannulation was not achieved by the junior endoscopists with ERCP experience of ≤100 procedures within 15–20 minutes, the senior endoscopists took over the procedure. If selective bile duct cannulation was not achieved within 5–10 minutes by endoscopists with ERCP experience of >100 procedures, the pancreatic guidewire method was used in patients with repetitive inadvertent pancreatic duct cannulation and the wire-guided cannulation was used in patients without inadvertent pancreatic duct cannulation. If selective bile duct cannulation using the conventional injection method, the pancreatic guidewire method, and the wire-guided cannulation was not achieved within 15–20 minutes by endoscopists with ERCP experience of >200 procedures, they used the precut technique under the supervision of senior endoscopists with ERCP experience of >500 procedures or senior endoscopists with ERCP experience of >500 procedures took over the procedure. If selective bile duct cannulation was not achieved within 15–20 minutes by endoscopists with ERCP experience of >500 procedures, one of the most experienced endoscopists took over the procedure. Operator change in the same patient was up to 2 times. The procedures were considered assigned to the endoscopists who started the cannulation. A low ERCP experience is associated with a short time of operator change to a senior endoscopist. Therefore, procedures of junior endoscopists with ERCP experience of ≤20 procedures were excluded from the analysis. Though the time limit for achieving selective bile duct cannulation could be changed depending on the reasons for ERCP and the patient's general condition, we set the limit to 60 minutes in most of the cases.

ERCP experience was defined as experience with ERCP-related procedures, including bile duct cannulation in patients with previous sphincterotomy, biliary stent exchange in patients with previous stent placement, and pancreatic duct cannulation in patients with pancreatic disease. Using the data from the electronic filing system of our institution, we were able to count the number of ERCP procedures performed by each endoscopist, because all endoscopists started their ERCP training at our institution.

A juxtapapillary diverticulum was defined as a duodenal diverticulum close to the papilla, with the oral protrusion of the papilla or the orifice of the papilla located at the rim of the diverticulum and the papilla located inside the diverticulum. Duodenal diverticula away from the papilla were not considered juxtapapillary diverticula. Among the juxtapapillary diverticula, if the orifice of the papilla was located just inside or outside of the borders of the diverticulum, it was defined as a papilla on the rim of the diverticulum.

The procedures with successful cannulation of the bile duct were divided into the following 2 groups: group L (cannulation time > 15 minutes) and group S (cannulation time ≤ 15 minutes). At our institution, as described above, if selective bile duct cannulation is not achieved within 15–20 minutes after junior endoscopists start the cannulation, senior endoscopists take over the procedure. Therefore, in case of operator change, the duration of the procedure exceeded 15 minutes and it was classified in group L. The endoscopists were classified into 6 groups according to the number of performed ERCP procedures: 21–100, 101–200, 201–300, 301–400, 401–500, and >500. The endoscopists could be changed from a group to another. For example, when the endoscopist with an ERCP experience of 100 procedures performed the next procedure, he or she was classified to the group with 101–200 procedures.

We compared the cannulation method and the proportion of PEP between groups L and S. PEP was diagnosed based on JPN diagnostic criteria [5]. We performed univariate analysis in order to identify the factors associated with a cannulation time of >15 minutes. We evaluated the association of the cannulation time with ERCP experience, diagnostic ERCP, acute cholangitis, juxtapapillary diverticula, papilla on the rim of the diverticulum, malignant biliary stricture, tumor invasion in the gastroduodenal tract, and bile duct stone impaction at the papilla. Baseline variables with *P* ≤ 0.25 in the univariate analysis were included in the multivariable models. The chi-square test, Fisher's exact test, or Student's *t*-test was used for univariate analysis, and logistic regression was used for multivariate analysis. The threshold for significance was *P* < 0.05. All statistical analyses were performed using SPSS version 22 (IBM, Armonk, NY, USA).

## 3. Results

After excluding the procedures performed by junior endoscopists with the ERCP experience of ≤20 procedures from 605 procedures, 556 procedures remained. Selective bile duct cannulation was successful in 531 procedures; the success rate was 95.5%. These procedures were divided into groups L and S according to the cannulation time. Group L included 166 procedures, and group S included 365 procedures.

### 3.1. Patient Characteristics, Cannulation Method, and PEP

The patient characteristics of both groups are presented in Table 1. No significant differences of age and sex were found between the groups. The proportion of conventional injection method was significantly lower in group L than that in group S. The proportion of PEP was significantly higher in group L than that in group S.

### 3.2. Factors Affecting Biliary Cannulation Time

In the univariate analysis, ERCP experience and malignant biliary stricture were found to significantly affect the cannulation time. ERCP experience was divided according to the number of procedures performed. We found no difference in the cannulation time between ERCP experience of >500 procedures and ERCP experience of 401–500 or 301–400 procedures. However, we found that ERCP experience of ≤300 procedures was significantly associated with a cannulation time of >15 minutes. Malignant biliary stricture was divided into 3 types: biliary cancer, pancreatic cancer, and the other cancerous lesions, such as metastatic lymph nodes. We found that malignant biliary stricture due to pancreatic cancer was significantly associated with a cannulation time of >15 minutes (Table 2).

Baseline variables with *P* ≤ 0.25 in the univariate analysis were included in the multivariable models. The experience of ≤300 procedures, diagnostic ERCP, juxtapapillary diverticula, malignant biliary stricture due to pancreatic cancer, malignant biliary stricture due to the other cancerous lesions, and bile duct stone impaction at the papilla were included in the multivariate analysis. In this analysis, ERCP experience of ≤300 procedures and malignant biliary stricture due to pancreatic cancer were found to be significantly associated with a cannulation time of >15 minutes (Table 3).

### 3.3. The Relationship between the ERCP Experience and the Cannulation Success Rate or the PEP Rate

We investigated the relationship between the ERCP experience and the bile duct cannulation success rate or the PEP rate, because the ERCP experience of ≤300 procedures was associated with a cannulation time of >15 minutes. We classified all 556 procedures for bile duct cannulation into the groups with the ERCP experience of 21–100, 101–200, 201–300, 301–400, 401–500, and >500 procedures and compared the cannulation success rate and the PEP rate between the groups. No significant differences in the final cannulation success rate were found between the groups. The cannulation success rate without operator change was less than 90% in the group of 21–100, 101–200, and 201–300 procedures, whereas more than 90% in the group of 301–400, 401–500, and >500 procedures (Table 4). The cannulation success rate without operator change was significantly lower in the group of 21–100 procedures than in that of >500 procedures. Although not significant, the group of 101–200 or 201–300 procedures tended to have a lower success rate of bile duct cannulation than that of >500 procedures (Table 5). No significant differences in PEP rate were detected between the groups (Table 4).

### 3.4. Reason for Long Cannulation Time in Patients with Malignant Biliary Stricture due to Pancreatic Cancer

We investigated the reason for long cannulation time in patients with malignant biliary stricture due to pancreatic cancer, because it was associated with a cannulation time of >15 minutes. We divided 58 procedures with malignant biliary stricture due to pancreatic cancer into groups L and S and compared bending of the lower bile duct to the pancreatic cancer, malignant stricture of the distal end of the lower bile duct, tumor invasion in the gastroduodenal tract, and poor endoscope operability in the duodenum between these groups. Poor endoscope operability was judged according to the medical records. The rate of all above 4 factors in group L was higher than that in group S, but the differences were not significant (Table 6).

## 4. Discussion

In this study, we analyzed the ERCP procedures for bile duct cannulation performed in patients with a native papilla and investigated the factors that affected the selective bile duct cannulation time. The results showed that ERCP experience of ≤300 procedures and malignant biliary stricture due to pancreatic cancer were significantly associated with a cannulation time of >15 minutes.

Several previous studies have evaluated the bile duct cannulation success rate and the number of ERCP procedures necessary for acquiring the skills for selective bile duct cannulation in patients with a native papilla [1, 2]. We believe that cannulation time is also important in addition to the success rate, because cannulation time of over 10–15 minutes has been reported to be a risk factor for post-ERCP pancreatitis (PEP) [3, 4]. To our best knowledge, no reports evaluated the relationship between the bile duct cannulation time and the ERCP experience. Ekkelenkamp et al. reported that the common bile duct cannulation success rate improved from 22% to 68% after 180 procedures. However, the number of procedures required to improve the success rate to 80% or 90% was not investigated [1]. Verma et al. reported that the cannulation success rate increased from 43% to 80% or more after 350–400 supervised procedures [2]. In our study, the ERCP experience of ≤300 procedures was associated with cannulation time of >15 minutes, and the rate of cannulation success without operator change was less than 90% in the group of 21–100, 101–200, and 201–300 procedures, whereas more than 90% in the group of 301–400, 401–500, and >500 procedures.

Few studies have performed a multivariate analysis to determine the relationship between bile duct cannulation difficulty and juxtapapillary diverticula, and these studies reported different results [6, 7]. Panteris et al. reported that a periampullary diverticulum might indicate easy cannulation [6]. In contrast, Zoepf et al. reported that cannulation of a papilla associated with a juxtapapillary diverticulum was significantly more difficult than cannulation in the absence of a diverticulum [7]. Regarding the papilla location in respect to the diverticulum and the effect on cannulation, Chang-Chien reported that the failure rate was higher when the papillary orifice was situated at the rim of the diverticulum than when the juxtapapillary diverticulum was situated beside the papilla and the papillary orifice was not at the rim of the diverticulum. However, the difference was not significant [8]. In our study, neither the presence of a papilla on the rim of the diverticulum nor the presence of a juxtapapillary diverticulum influenced the bile duct cannulation time.

No reports of the relationship between the malignant biliary stricture and the difficulty of bile duct cannulation are available. In our study, malignant biliary stricture due to pancreatic cancer was associated with cannulation time of >15 minutes. The reason for this result remains unclear, although from data reported in Table 6, poor endoscope operability in the duodenum, a parameter that is difficult to be judged objectively, may be one of the reasons.

Several limitations of this study should be acknowledged. Multivariate analysis can reduce the bias related to the investigated differences between groups L and S, but biases still exist from uninvestigated differences, such as anatomical constraints or papillary spasm. In this study, the first-line method for bile duct cannulation was conventional injection method, but whether the same results can be obtained when using wire-guided cannulation as the first-line cannulation method remains unclear. At our institution, ERCP training is performed along with esophagogastroduodenoscopy, colonoscopy, endoscopic ultrasonography, endoscopic treatments, including mucosal resection and submucosal dissection for tumors, and other similar procedures. The number of endoscopic procedures, excluding ERCP, performed by each endoscopist could not be investigated, and it is unclear whether the ERCP experience of 300 procedures reported by our study can be extended to trainees who undergo a different training method. In our study, the rate of PEP was significantly higher in group L than that in group S, but did not differ significantly by the difference of ERCP experience. It is difficult to discuss about PEP from these results, because various factors have been shown to influence the occurrence of PEP [9].

However, we believe that our investigation of the factors affecting the bile duct cannulation time is meaningful. The ERCP experience of 300 procedures can be regarded as the required number of procedures for acquiring the skills for selective bile duct cannulation, because an ERCP experience of ≤300 procedures was not only associated with the long cannulation time but also tended to have lower success rate of the bile duct cannulation. In patients with malignant biliary stricture due to pancreatic cancer, we should decide earlier operator change to senior endoscopists or earlier use of the precut technique.

In conclusion, our analysis demonstrated that bile duct cannulation time of >15 minutes was associated with the ERCP experience of ≤300 procedures and the malignant biliary stricture due to pancreatic cancer.

## Figures and Tables

**Table 1:** Clinical characteristics of the patients, applied cannulation method, and PEP incidence.

	Group L (*n* = 166)	Group S (*n* = 365)	Two-tailed *p* value	Odds ratio	95% CI
Mean age (SD), years	69.3 (13.3)	70.1 (14.5)	0.533^a^	—	−3.4337–1.7785
Sex (male), *n*	94	211	0.798^b^	0.953	0.658–1.380
Cannulation method, *n*	Injection 86	Injection 342	0.000^b^	—	—
	WGC 18	WGC 12			
	PGW 40	PGW 10			
	Precut 22	Precut 1			
PEP, *n* (%)	26 (15.6)	7 (1.9)	0.000^b^	9.498	4.031–22.381

CI: confidence interval; SD: standard deviations; WGC: wire-guided cannulation; PGW: pancreatic guidewire method; Precut: percutaneous; PEP: post-ERCP pancreatitis; ^a^Student's *t*-test; ^b^Chi-square test.

**Table 2:** Univariate analysis of the factors affecting biliary cannulation time.

Factor	Group L (*n* = 166)	Group S (*n* = 365)	Two-tailed *p* value	Odds ratio	95% CI
ERCP experience, *n*			0.013^a^		
21–100	39	50	0.000^a^	3.018	1.622–5.616
101–200	49	94	0.016^a^	2.017	1.136–3.581
201–300	33	69	0.049^a^	1.851	0.995–3.434
301–400	16	47	0.458^a^	1.317	0.635–2.732
401–500	6	16	0.571^b^	1.451	0.511–4.123
>500	23	89		1	
Diagnostic ERCP, *n*	23	66	0.227^a^	0.729	0.435–1.219
Acute cholangitis, *n*	17	49	0.303^a^	0.736	0.410–1.321
Juxtapapillary diverticula, *n*	36	101	0.144^a^	0.724	0.469–1.118
Papilla on the rim of the diverticulum, *n*	8	13	0.491^a^	1.371	0.557–3.374
Malignant biliary stricture, *n*	50	87	0.037^a^		
Biliary cancer, *n*	17	48	0.572^a^	0.843	0.465–1.527
Pancreatic cancer, *n*	27	32	0.013^a^	2.008	1.151–3.501
The other cancerous lesions, *n*	6	7	0.224^b^	2.039	0.671–6.199
None, *n*	116	276		1	
Tumor invasion in the gastroduodenal tract, *n*	6	13	0.976^a^	1.015	0.379–2.720
Bile duct stone impaction at the papilla, *n*	5	26	0.061^a^	0.405	0.153–1.074

ERCP: endoscopic retrograde cholangiopancreatography; CI: confidence interval; ^a^Chi-square test; ^b^Fisher's exact test.

**Table 3:** Multivariate analysis of factors related to the L group.

Factor	Two-tailed *p* value	Odds ratio	95% CI
ERCP experience of ≤300 procedures	0.001	2.080	1.377–3.142
Diagnostic ERCP	0.349	0.775	0.454–1.322
Juxtapapillary diverticula	0.353	0.808	0.516–1.266
Malignant biliary stricture due to pancreatic cancer	0.028	1.912	1.072–3.412
Malignant biliary stricture due to the other cancerous lesions	0.146	2.329	0.745–7.276
Bile duct stone impaction at the papilla	0.067	0.395	0.147–1.065

ERCP: endoscopic retrograde cholangiopancreatography; CI: confidence interval.

**Table 4:** The relationship between the ERCP experience and cannulation success rate or PEP.

	ERCP experience						Two-tailed *p* value
	21–100 (*n* = 92)	101–200 (*n* = 150)	201–300 (*n* = 109)	301–400 (*n* = 65)	401–500 (*n* = 23)	>500 (*n* = 117)	
Final cannulation success, *n* (%)	89 (96.7)	143 (95.3)	102 (93.5)	63 (96.9)	22 (95.6)	112 (95.7)	0.901^a^
Cannulation success without operator change, *n* (%)	55 (59.7)	129 (86.0)	94 (86.2)	61 (93.8)	22 (95.6)	109 (93.1)	0.000^a^
PEP, *n* (%)	5 (5.4)	10 (6.6)	5 (4.5)	5 (7.6)	3 (13.0)	8 (6.8)	0.756^a^

ERCP: endoscopic retrograde cholangiopancreatography; PEP: post-ERCP pancreatitis; ^a^Chi-square test.

**Table 5:** The relationship between the ERCP experience and cannulation success rate without operator change.

	Cannulation success without operator change, *n* (%)	Two-tailed *p* value	Odds ratio	95% CI
ERCP experience		0.000^a^		
21–100	55 (59.7)	0.000^a^	0.109	0.048–0.250
101–200	129 (86.0)	0.082^a^	0.473	0.201–1.117
201–300	94 (86.2)	0.085^a^	0.460	0.187–1.133
301–400	61 (93.8)	1.000^b^	1.119	0.324–3.870
401–500	22 (95.6)	1.000^b^	1.615	0.192–13.570
>500	109 (93.1)		1	

ERCP: endoscopic retrograde cholangiopancreatography; CI: confidence interval; ^a^Chi-square test; ^b^Fisher's exact test.

**Table 6:** The reason for long cannulation time in patients with malignant biliary stricture due to pancreatic cancer.

	Group L (*n* = 26)	Group S (*n* = 32)	Two-tailed *p* value	Odds ratio	95% CI
Bending of the lower bile duct to the pancreatic cancer, *n* (%)	8 (30.7)	5 (15.6)	0.169^a^	2.400	0.676–8.517
Malignant stricture of the distal end of the lower bile duct, *n* (%)	7 (26.9)	6 (18.7)	0.458^a^	1.596	0.462–5.520
Tumor invasion in the gastroduodenal tract, *n* (%)	7 (26.9)	7 (21.8)	0.655^a^	1.316	0.394–4.393
Poor endoscope operability in the duodenum, *n* (%)	8 (30.7)	6 (18.7)	0.287^a^	1.926	0.570–6.505

CI: confidence interval; ^a^Chi-square test.
